# Transmission of Legionnaires’ Disease through Toilet Flushing

**DOI:** 10.3201/eid2607.190941

**Published:** 2020-07

**Authors:** Jeanne Couturier, Christophe Ginevra, Didier Nesa, Marine Adam, Cyril Gouot, Ghislaine Descours, Christine Campèse, Giorgia Battipaglia, Eolia Brissot, Laetitia Beraud, Anne-Gaëlle Ranc, Sophie Jarraud, Frédéric Barbut

**Affiliations:** Hôpital Saint-Antoine, Paris, France (J. Couturier, D. Nesa, M. Adam, C. Gouot, G. Battipaglia, E. Brissot, F. Barbut);; Faculté de Pharmacie de Paris, Université de Paris, France (J. Couturier, F. Barbut);; Centre National de Référence des Légionelles, Lyon, France (C. Ginevra, G. Descours, L. Beraud, A.-G. Ranc, S. Jarraud);; Université Claude Bernard Lyon 1, Villeurbanne, France (C. Ginevra, G. Descours, L. Beraud, A.-G. Ranc, S. Jarraud);; Santé Publique France, Saint-Maurice, France (C. Campèse);; Sorbonne Université, Paris, France (E. Brissot)

**Keywords:** Bacteria, healthcare-associated infections, Legionnaires’ disease, nosocomial infections, phylogeny, pneumophilia, respiratory infections, toilet, transmission, Legionella pneumophila

## Abstract

We describe 2 cases of healthcare-associated Legionnaires’ disease in patients in France hospitalized 5 months apart in the same room. Whole-genome sequencing analyses showed that clinical isolates from the patients and isolates from the room’s toilet clustered together. Toilet contamination by *Legionella pneumophila* could lead to a risk for exposure through flushing.

*Legionella pneumophila* is a gram-negative bacterium usually found in small amounts in water in both nature and built environments. In larger amounts, it can be responsible for a severe pneumonia known as Legionnaires’ disease (LD). Transmission usually occurs when someone inhales contaminated aerosols from showers, cooling towers, faucets, or fountains. Person-to-person transmission is extremely rare (*1*). Researchers have shown evidence of a variety of other uncommon sources of contamination, such as windshield washer fluid (*2*) or dental unit waterlines (*3*). LD transmission through flushing toilets has also been suspected (*4*) but not demonstrated. We report 2 cases of LD in immunocompromised patients in France, potentially caused by *L. pneumophila* transmission through flushing toilets. 

## The Study 

In the first case, an 18-year-old woman who had undergone an allogeneic bone marrow transplant for acute myeloid leukemia in February 2014 in a hospital hematology unit in France was hospitalized in December 2015 in the same unit for 9 days. At that time, she received immunosuppressive agents (steroids, cyclosporine A, and ruxolitinib) for a chronic graft-versus-host disease. She was readmitted to the hematology unit with fever (38.5°C), shivering, and dyspnea 6 days after discharge. Six days later, she was transferred to the medical intensive care unit (MICU) of the same hospital with fever (40°C), bilateral pneumonia, and respiratory and renal failure. The result from a *L. pneumophila* urinary antigen test (BinaxNOW Legionella Urinary Antigen EIA kit; Abbott, https://www.abbott.com) was positive at the time of MICU admission, leading clinicians to consider this LD case to be probably healthcare associated. Physicians successfully treated the patient with spiramycin and levofloxacin, and she was discharged from the MICU after 8 days. 

In the second case, a 51-year-old man was admitted to the same hematology unit in May 2016 for an autologous transplant for a recurrent Hodgkin lymphoma. A persistent fever (38.8°C) appeared on the 12th day after admission and a computed tomography scan of the chest showed a multifocal consolidation. The result from a *L. pneumophila* urinary antigen test (BinaxNOW) on day 22 was positive; therefore, this LD case was considered to be definitely healthcare associated. The patient’s condition suddenly worsened, and he was transferred to the MICU, where he rapidly recovered after doctors treated him with spiramycin and levofloxacin. 

For both patients, a bronchoalveolar lavage procedure found *L. pneumophila* bacteria from serogroup 1 (LP1), pulsed-field gel electrophoresis pulsotype Paris, sequence type (ST) 1, monoclonal antibody subgroup Philadelphia. Both patients had been hospitalized in the same room (room 1) of the hematology unit, 5 months apart. Air filtration systems with HEPA filters were used to control the environment of the unit. Water from the sink in each room and from the shower, shared by all of the unit’s patients, was filtered through 0.1-µm pore filters. Both patients were provided only bottled water and did not take showers during their hospital stay. In addition, there was no cooling tower within the hospital. 

We sampled and analyzed the potential sources of exposure in accordance with the NF T 90-431 standard (Association Française de Normalisation, https://www.afnor.org) for counting *Legionella* spp., after the second LD case. In brief, we sampled 500 mL of water in sterile vials containing 20 mg sodium thiosulfate. First, we inoculated 0.2 mL of water on GVPC (glycine, vancomycin, polymyxin, cycloheximide) plates (Oxoid France, http://www.oxoid.com/fr/blue/). Then, we filtered 10 mL and 100 mL of water through 0.2-µm pore polycarbonate membranes placed on GVPC media. We incubated plates at 36°C (+ 2°C) for 8–11 days. We subcultured suspicious colonies on buffered charcoal yeast extract media with and without cystein and identified them by latex agglutination (Oxoid France). 

We found no *L. pneumophila* in the hot water from the shared shower (temperature 26.0°C, chlorine 1.12 mg/L), nor in the hot or cold water from the sink in room 1 (temperatures 24°C and 25°C, chlorine 1.11 mg/L and 0 mg/L, respectively). However, sampling of the water from the toilet bowl in room 1 showed contamination (1,100 CFU/L LP1, with a temperature of 22.0°C and no chlorine). LP1 was also in the toilet bowl of the adjacent room (room 2) (100 CFU/L, temperature 24.4°C), the hot water from the sink in the nurses’ office (10 CFU/L, temperature 49.8°C, chlorine 0.77 mg/L), and the cold-water inlet of the building (20 CFU/L, temperature 15.0°C). Contamination in the cold-water inlet (20 CFU/L, temperature 9.0°C) had also been detected in March 2016. Tests of samples of toilet water from tanks in nearby rooms did not show any contamination. 

The room was closed, and the toilet was disinfected daily with bleach. The toilet water in the room was monitored through iterative testing; results were negative from 10 successive samples tested between June 2016 and November 2017. To determine the extent of such contamination, 29 toilets in 5 different hospital buildings were analyzed. All samples were negative, suggesting that *L. pneumophila* contamination of toilet water was not common. 

We used Nextera XT technology (Illumina, https://www.illumina.com) for whole-genome sequencing of 2 clinical and 10 environmental LP1 strains and deposited raw reads into the European Nucleotide Archive (study accession no. PRJEB32615). We used SPAdes to assemble genomes (*5*) and mompS tool to extract STs from the WGS data (*6*). All 12 strains belonged to ST1. We analyzed these 12 strains in more depth by comparing their genomes to 27 other epidemiologically unrelated LP1 ST1 genomes from France available in the Sequence Read Archive database (Figure; Appendix). We also performed phylogenetic analyses on this genome’s dataset, as described by David et al. (*7*). 

**Figure:**
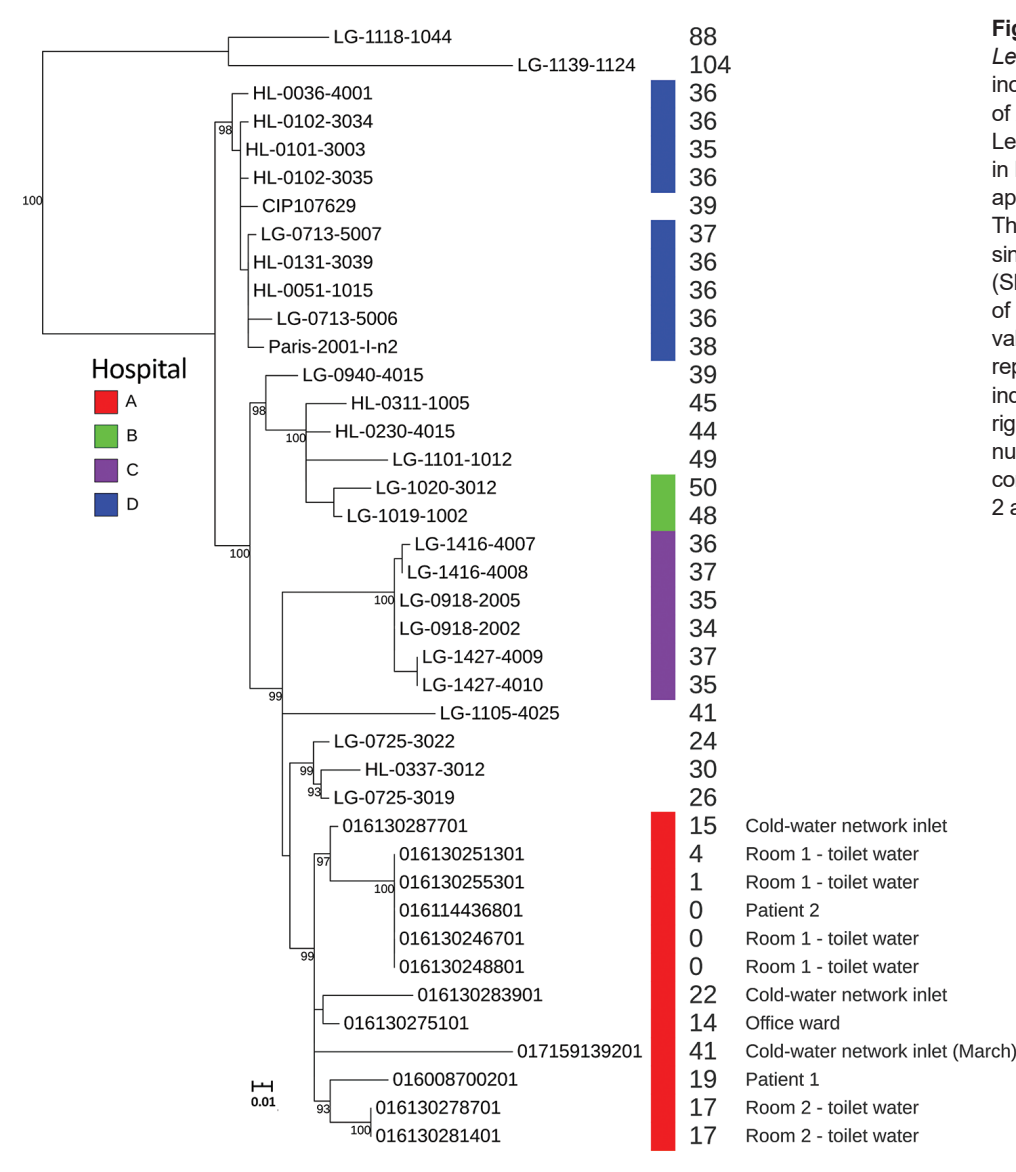
Maximum-likelihood tree of 39 *Legionella pneumophila* ST1 isolates, including isolates from investigation of 2 cases of healthcare-associated Legionnaires’ disease in patients in France hospitalized 5 months apart in the same room (red bar). The tree was constructed using 258 single-nucleotide polymorphisms (SNPs) identified after the removal of recombination events. Bootstrap values were calculated from 500 replicates; only values >90 were indicated on the tree. Values at the right side of the tree represent the number of SNPs between the genome compared with the genome of patient 2 after recombination removal.

The 2 clinical isolates were nested within and thus derived from the clade of isolates sampled from the hospital water network. All 12 strains, including the strains isolated in the cold-water inlet, were part of a cluster sharing the same most recent common ancestor (Figure). We observed no difference in single-nucleotide polymorphisms (SNPs) between the 2 isolates from the toilet water from the patients’ room and the isolate of the second patient (Figure). The strains present in the toilet water were identical or closely related to the strains infecting the patients, and no other potential contamination source was identified, strongly suggesting that the toilet water was the contamination source. The few differences in SNPs between the first clinical isolate and the isolates found in the environment (0–23 SNPs; see Appendix Table) could be explained by diversity of the ST1 population in the water network or the micro-evolution of the environmental LP1 population during the 5 months between the 2 LD cases (*7*). 

## Conclusions

We describe 2 cases in which LD was probably caused by *L. pneumophila* transmitted through contaminated toilet water that became aerosolized during flushing. We reached this conclusion because we found little to no detectable difference between whole genomes in isolates obtained from 2 patients hospitalized 5 months apart in the same room and those from the toilet in that room. The other commonly suspected sources, in this case the shower and the sink, tested negative for *L. pneumophila*. 

This investigation suggests that transmission of *L. pneumophila* through toilet flushing should be considered when investigating a LD case. However, as previously suggested, there remains a need for a laboratory-based study to explore whether flushing toilets can generate and spread contaminated aerosols (*8*,*9*). 

Appendix Additional information on transmission of Legionnaires’ disease through toilet flushing.
