# Supplementary material for: Transmission of Legionnaires’ Disease through Toilet Flushing
Source: Emerg Infect Dis. 2020 Jul;26(7):1526–8. doi: 10.3201/eid2607.190941 (PMC7323554; doi:10.3201/eid2607.190941)
Supplement: Appendix — Additional information on transmission of Legionnaires’ disease through toilet flushing. [file 19-0941-Techapp-s1.pdf]

Article DOI: https://doi.org/10.3201/eid2607.190941

Transmission of Legionnaires' Disease through Toilet Flushing

Appendix

Phylogenetic Analyses

The genomes were compared by mapping the reads against the reference genome NC_006368.1 of the strain Paris CIP107629. The reads were mapped using snippy (https://github.com/tseemann/snippy) with a minimum depth coverage of 10x. A total of 3,087 core SNPs were identified. SNPs inferred as being recombination events were removed from the full alignment generated by snippy using Gubbins (*1*). The remaining 258 SNPs were used to construct an unrooted maximum likelihood phylogenetic tree based on the GTRGAMMA model using RAxML https://github.com/stamatak/standard-RAxML (*2*). A total of 500 bootstraps were performed to assess the robustness of the tree (Figure). An all-against-all pairwise identity analysis was also performed using the alignment generated by Gubbins. The resulting distance matrix is available in the table below.

References

1. Croucher NJ, Page AJ, Connor TR, Delaney AJ, Keane JA, Bentley SD, et al. Rapid phylogenetic analysis of large samples of recombinant bacterial whole genome sequences using Gubbins. Nucleic Acids Res. 2015;43:e15. PubMed https://doi.org/10.1093/nar/gku1196

2. Stamatakis A. RAxML version 8: a tool for phylogenetic analysis and post-analysis of large phylogenies. Bioinformatics. 2014;30:1312–3. PubMed https://doi.org/10.1093/bioinformatics/btu033

Isolates ID	016008700201	016114436801	016130246701	016130248801	016130251301	016130255301	016130275101	016130278701	016130281401	016130283901	016130287701	017159139201	LG-1427-4009	LG-1427-4010	LG-1416-4007	LG-0918-2005	LG-0918-2002	LG-1416-4008	LG-1019-1002	LG-1020-3012
016008700201	0	19	19	19	23	20	13	12	12	21	18	39	36	34	35	34	33	36	47	49
016114436801	19	0	0	0	4	1	14	17	17	22	15	41	37	35	36	35	34	37	48	50
016130246701	19	0	0	0	4	1	14	17	17	22	15	41	37	35	36	35	34	37	48	50
016130248801	19	0	0	0	4	1	14	17	17	22	15	41	37	35	36	35	34	37	48	50
016130251301	23	4	4	4	0	3	18	21	21	25	11	40	35	33	34	33	32	35	46	48
016130255301	20	1	1	1	3	0	15	18	18	22	14	40	38	36	37	36	35	38	49	51
016130275101	13	14	14	14	18	15	0	11	11	14	13	33	31	29	30	29	26	31	41	43
016130278701	12	17	17	17	21	18	11	0	0	19	16	38	34	32	33	32	31	34	45	47
016130281401	12	17	17	17	21	18	11	0	0	19	16	38	34	32	33	32	31	34	45	47
016130283901	21	22	22	22	25	22	14	19	19	0	20	42	39	37	38	37	36	39	50	52
016130287701	18	15	15	15	11	14	13	16	16	20	0	34	28	26	27	26	25	28	37	39
017159139201	39	41	41	41	40	40	33	38	38	42	34	0	49	49	50	47	48	47	56	58
LG-1427-4009	36	37	37	37	35	38	31	34	34	39	28	49	0	2	5	3	5	4	43	45
LG-1427-4010	34	35	35	35	33	36	29	32	32	37	26	49	2	0	3	2	3	4	43	45
LG-1416-4007	35	36	36	36	34	37	30	33	33	38	27	50	5	3	0	3	4	3	44	46
LG-0918-2005	34	35	35	35	33	36	29	32	32	37	26	47	3	2	3	0	3	2	41	43
LG-0918-2002	33	34	34	34	32	35	26	31	31	36	25	48	5	3	4	3	0	5	43	45
LG-1416-4008	36	37	37	37	35	38	31	34	34	39	28	47	4	4	3	2	5	0	41	43
LG-1019-1002	47	48	48	48	46	49	41	45	45	50	37	56	43	43	44	41	43	41	0	6
LG-1020-3012	49	50	50	50	48	51	43	47	47	52	39	58	45	45	46	43	45	43	6	0
HL-0131-3039	35	36	36	36	34	37	29	33	33	38	27	45	31	31	32	29	31	29	30	33
LG-0713-5006	35	36	36	36	34	37	28	33	33	38	27	50	35	33	34	33	30	35	37	40
HL-0051-1015	35	36	36	36	34	37	29	33	33	38	27	45	31	31	32	29	31	29	30	33
HL-0102-3034	35	36	36	36	34	37	29	33	33	38	27	45	31	31	32	29	31	29	30	33
HL-0102-3035	35	36	36	36	34	37	29	33	33	38	27	45	31	31	32	29	31	29	30	33
Paris-2001-I-n2	37	38	38	38	36	39	31	35	35	40	29	47	33	33	34	31	33	31	32	35
LG-0713-5007	36	37	37	37	35	38	30	34	34	39	28	46	32	32	33	30	32	30	31	34
HL-0101-3003	34	35	35	35	33	36	27	32	32	37	26	43	30	30	31	28	29	28	30	33
HL-0036-4001	35	36	36	36	34	37	29	33	33	38	27	45	31	31	32	29	31	29	30	33
LG-0725-3022	23	24	24	24	22	25	18	21	21	26	15	37	23	22	23	20	22	22	33	35
LG-0725-3019	25	26	26	26	24	27	20	23	23	28	17	36	23	23	24	21	24	21	32	34
HL-0230-4015	43	44	44	44	42	45	37	41	41	46	35	52	39	39	40	37	39	37	14	16
HL-0311-1005	44	45	45	45	43	46	38	42	42	47	36	53	40	40	39	38	40	38	15	17
HL-0337-3012	29	30	30	30	28	31	24	27	27	32	21	40	27	27	28	25	28	25	36	38
LG-0940-4015	38	39	39	39	37	40	32	36	36	41	30	47	34	34	35	32	34	32	19	22
LG-1101-1012	48	49	49	49	47	50	42	46	46	51	40	57	44	44	45	42	44	42	19	20
LG-1105-4025	40	41	41	41	39	42	35	38	38	43	32	51	36	36	37	34	37	34	43	45
LG-1118-1044	87	88	88	88	86	89	81	85	85	89	79	96	82	82	81	80	82	79	79	82
LG-1139-1124	103	104	104	104	102	105	97	101	101	105	95	112	98	98	97	96	98	95	92	96
CIP107629	38	39	39	39	35	38	32	36	36	40	27	46	34	34	35	32	34	32	32	35

Isolates ID	HL-0131-3039	LG-0713-5006	HL-0051-1015	HL-0102-3034	HL-0102-3035	Paris-2001-I-n2	LG-0713-5007	HL-0101-3003	HL-0036-4001	LG-0725-3022	LG-0725-3019	HL-0230-4015	HL-0311-1005	HL-0337-3012	LG-0940-4015	LG-1101-1012	LG-1105-4025	LG-1118-1044	LG-1139-1124	CIP107629
016008700201	35	35	35	35	35	37	36	34	35	23	25	43	44	29	38	48	40	87	103	38
016114436801	36	36	36	36	36	38	37	35	36	24	26	44	45	30	39	49	41	88	104	39
016130246701	36	36	36	36	36	38	37	35	36	24	26	44	45	30	39	49	41	88	104	39
016130248801	36	36	36	36	36	38	37	35	36	24	26	44	45	30	39	49	41	88	104	39
016130251301	34	34	34	34	34	36	35	33	34	22	24	42	43	28	37	47	39	86	102	35
016130255301	37	37	37	37	37	39	38	36	37	25	27	45	46	31	40	50	42	89	105	38
016130275101	29	28	29	29	29	31	30	27	29	18	20	37	38	24	32	42	35	81	97	32
016130278701	33	33	33	33	33	35	34	32	33	21	23	41	42	27	36	46	38	85	101	36
016130281401	33	33	33	33	33	35	34	32	33	21	23	41	42	27	36	46	38	85	101	36
016130283901	38	38	38	38	38	40	39	37	38	26	28	46	47	32	41	51	43	89	105	40
016130287701	27	27	27	27	27	29	28	26	27	15	17	35	36	21	30	40	32	79	95	27
017159139201	45	50	45	45	45	47	46	43	45	37	36	52	53	40	47	57	51	96	112	46
LG-1427-4009	31	35	31	31	31	33	32	30	31	23	23	39	40	27	34	44	36	82	98	34
LG-1427-4010	31	33	31	31	31	33	32	30	31	22	23	39	40	27	34	44	36	82	98	34
LG-1416-4007	32	34	32	32	32	34	33	31	32	23	24	40	39	28	35	45	37	81	97	35
LG-0918-2005	29	33	29	29	29	31	30	28	29	20	21	37	38	25	32	42	34	80	96	32
LG-0918-2002	31	30	31	31	31	33	32	29	31	22	24	39	40	28	34	44	37	82	98	34
LG-1416-4008	29	35	29	29	29	31	30	28	29	22	21	37	38	25	32	42	34	79	95	32
LG-1019-1002	30	37	30	30	30	32	31	30	30	33	32	14	15	36	19	19	43	79	92	32
LG-1020-3012	33	40	33	33	33	35	34	33	33	35	34	16	17	38	22	20	45	82	96	35
HL-0131-3039	0	7	0	2	2	2	1	2	4	21	20	28	27	24	19	32	32	61	77	5
LG-0713-5006	7	0	7	9	9	9	8	7	11	24	26	35	34	30	26	39	38	68	84	12
HL-0051-1015	0	7	0	2	2	2	1	2	4	21	20	28	27	24	19	32	32	61	77	5
HL-0102-3034	2	9	2	0	2	4	3	2	4	21	20	28	27	24	19	32	32	61	77	5
HL-0102-3035	2	9	2	2	0	4	3	2	4	21	20	28	27	24	19	32	32	61	77	5
Paris-2001-I-n2	2	9	2	4	4	0	3	4	6	23	22	30	29	26	21	34	34	63	79	7
LG-0713-5007	1	8	1	3	3	3	0	3	5	22	21	29	28	25	20	33	33	62	78	6
HL-0101-3003	2	7	2	2	2	4	3	0	4	20	19	28	27	23	19	32	31	61	77	5
HL-0036-4001	4	11	4	4	4	6	5	4	0	21	20	28	27	24	19	32	32	61	77	7
LG-0725-3022	21	24	21	21	21	23	22	20	21	0	5	29	30	9	24	34	26	73	89	24
LG-0725-3019	20	26	20	20	20	22	21	19	20	5	0	28	29	6	23	33	25	72	88	23
HL-0230-4015	28	35	28	28	28	30	29	28	28	29	28	0	11	32	17	17	41	77	90	31
HL-0311-1005	27	34	27	27	27	29	28	27	27	30	29	11	0	33	16	17	42	74	90	30
HL-0337-3012	24	30	24	24	24	26	25	23	24	9	6	32	33	0	27	37	29	75	92	27
LG-0940-4015	19	26	19	19	19	21	20	19	19	24	23	17	16	27	0	21	35	68	84	22
LG-1101-1012	32	39	32	32	32	34	33	32	32	34	33	17	17	37	21	0	46	81	97	35
LG-1105-4025	32	38	32	32	32	34	33	31	32	26	25	41	42	29	35	46	0	84	100	35
LG-1118-1044	61	68	61	61	61	63	62	61	61	73	72	77	74	75	68	81	84	0	44	64
LG-1139-1124	77	84	77	77	77	79	78	77	77	89	88	90	90	92	84	97	100	44	0	80
CIP107629	5	12	5	5	5	7	6	5	7	24	23	31	30	27	22	35	35	64	80	0

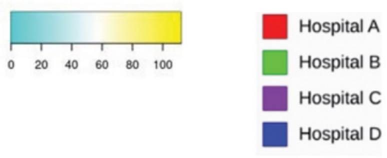

Appendix Figure. Distance matrix from the all-against-all pairwise identity analysis of SNPs between isolates after recombination events removal using Gubbins. Colors of the firsts raw and column represent the different hospitals. Colors gradient represents a heat map according to SNPs number.
